# The Role of Vertebral Morphometry in the Pathogenesis of Degenerative Lumbar Spinal Stenosis

**DOI:** 10.1155/2021/7093745

**Published:** 2021-09-04

**Authors:** Janan Abbas, Natan Peled, Israel Hershkovitz, Kamal Hamoud

**Affiliations:** ^1^Department of Anatomy and Anthropology, Sackler Faculty of Medicine, Tel Aviv University, Tel Aviv 6997801, Israel; ^2^Department of Physical Therapy, Zefat Academic College, Zefat, 13206, Israel; ^3^Department of Radiology, Carmel Medical Center, Haifa 3436212, Israel; ^4^Azrieli Faculty of Medicine, Bar-Ilan University, Safed 1311502, Israel; ^5^Department of Orthopaedic Surgery, The Baruch Padeh Poriya Medical Center, Tiberias 1520800, Israel

## Abstract

The aim of the current study was to establish whether the vertebral morphometry (e.g., vertebral body width and spinal canal diameters) is associated with degenerative lumbar spinal stenosis (DLSS). A retrospective computerized tomography (CT) study from L1 to L5 for two sample populations was used. The first included 165 participants with symptomatic DLSS (sex ratio 80 M/85F), and the second had 180 individuals from the general population (sex ratio: 90 M/90F). Vertebral body length (VL) and width (VW) were significantly greater in the stenosis males and females compared to their counterparts in the control. The mean VL in the stenosis males was 31.3 mm at L1, 32.6 mm at L2, 34 mm at L3, 34.1 mm at L4, and 34.5 at L5 compared to 29.9 mm, 31.3 mm, 32.6 mm, 32.8 mm, and 32.9, respectively, in the control group (*P* ≤ 0.003). Additionally, the bony anterior-posterior (AP) canal diameters and cross-sectional area (CSA) were significantly smaller in the stenosis group compared to the control. The mean AP canal values in the stenosis males were 17.8 mm at L1, 16.6 mm at L2, 15.4 mm at L3, 15.6 mm at L4, and 16.1 at L5 compared to 18.7, 17.8, 16.9, 17.6, and 18.8, respectively, in the control group. Vertebral length (OR-1.273 to 1.473; *P* ≤ 0.002), AP canal diameter (OR-0.474 to 0.664; *P* ≤ 0.007), and laminar inclination (OR-0.901 to 0.856; *P* ≤ 0.025) were significantly associated with DLSS. Our study revealed that vertebral morphometry has a role in DLSS development.

## 1. Introduction

Lumbar spinal stenosis is defined as an encroachment of the neurovascular elements and can be developmental (congenital) and acquired [1]. Congenital stenosis is multileveled segments that appears in the younger age (30 to 40 years), whereas the acquired degenerative is by far the most common type usually single level that is related to the advanced age of 50th to 60th years [2, 3]. Degenerative lumbar spinal stenosis (DLSS) is typically associated with degeneration of the intervertebral disc anteriorly, as well as 2-facet joints and ligamentum flavum posteriorly [3–5]. More so, DLSS is classified into central, lateral recess, or foraminal in which different symptoms may present and various surgical treatment options needed accordingly [6]. The main symptoms for this phenomenon are neurogenic claudication and radicular pain [7].

It is well accepted that DLSS typically associates with the available area for the dural sac rather than the bony spinal canal diameters [8, 9]. However, there is some evidence that correlates this phenomenon with the vertebral bony morphology besides to the degenerative process in the spine segment [10, 11]. For example, it has been previously reported that subjects with DLSS have greater vertebral body size than the controls and the pedicle width increases the risk for DLSS development. Others have also showed that various spine pathologies, such as osteoarthritis, spondylolisthesis, and Scheuermann's disease, could be related to the vertebral body morphometry [12–15]. Accordingly, we hypothesize that vertebral morphometry may have a role in the pathogenesis of DLSS.

The aim of this study was to establish whether the vertebral morphometry (e.g., vertebral body size and lamina inclination) is associated with DLSS.

## 2. Materials and Methods

### 2.1. Study Design

This is a retrospective study including two groups of participants that were enrolled between 2008 and 2012 years, following the project of Abbas et al., 2020 [16]. The first group included 165 individuals with DLSS-related symptoms (sex ratio: 80M/85F), and age range was 40-88 years. All individuals had stenosis at one or more levels (cross-section area of the dural sac < 100 mm^2^) [8], as a result of the degeneration of the spine segment (e.g., facet hypertrophy, thickening of LF). The diagnostic criteria for DLSS in each individual included a combination of clinical symptoms and signs together with the computerized tomography (CT) findings [7]. The clinical criteria for inclusion were based on Spine Patient Outcomes Research Trail (SPORT) [17, 18] and was done by one of the coauthor (KH) who is a spine surgeon. Individuals under the age of 40 years as well as those with congenital stenosis (anterior posterior diameter of the bony canal < 12 mm) [19], fractures, spondylolysis, tumors, Paget's disease, steroid treatment, severe lumbar scoliosis (>20 degrees), and iatrogenic conditions were excluded from this study. In the second group, the control included 180 patients without spinal stenosis related symptoms who were referred to the Department of Radiology, Carmel Medical Center, Haifa, Israel, for abdominal CT scans due to renal colic symptoms/or abdominal pain. The age range was 40-99 years with sex ratio: 90M/90F. A high-resolution CT image (Brilliance 64, Philips Medical Systems, voltage 120 kV, slice thickness 0.9–3 mm, current 150–570 mA) was utilized which enabled scan processing in all planes. All the CT images for both groups were taken in the supine position with extended knees. The ethical committee of the Carmel Medical Center (0083-07-CMC) approved this research.

ALL measurements were taken in bone window from L1 to L5. Vertebral body and canal parameter assessment wereperformed based on the study of Perry el al. (1987) and adapted to CT images [20].

### 2.2. Vertebral Body Length

**Figure 1:**
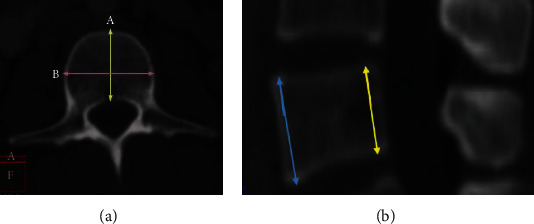
Measurements of vertebral body diameters: body length and width (a) and body heights (b).

(VL) and width (VW) were measured in the axial plane, at the mid vertebral height. VL was defined as the longest line that connects the anterior and posterior vertebral body borders, whereas the VW is the longest line that connects the right and left vertebral body borders (Figure 1).

### 2.3. Vertebral Body Height

(VH) was measured in the midsagittal plane, at its anterior (AH), and posterior aspects (pH). AH was measured as the distance between the anterosuperior and anteroinferior corners of the vertebral body, whereas pH was considered as the distance between the posterosuperior and posteroinferior corners of the vertebral body (Figure 1). The ratio of vertebral body beveling was defined as the AH/pH × 100%.

### 2.4. Bony Canal Diameters

**Figure 2:**
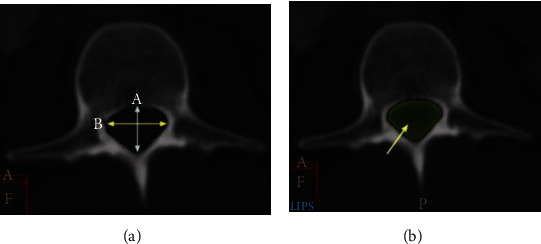
Measurements of bony canal diameters: anterior-posterior diameter, mediolateral diameter (a), and cross-section area of bony canal (b).

The anterior-posterior (AP), mediolateral (ML), and cross-section area (CSA) were measured in the axial plane at the midvertebral body, at the level of the basivertebral foramen. AP was measured as the longest line that extends from the posterior vertebral border to the posterior canal border, whereas ML defines as the longest line that connects the medial border of the two pedicles. Bony CSA was the area of the bony spinal canal, delineated by the posterior vertebral cortex, the medial borders of the two pedicles, and the anterior border of the neural arch (Figure 2).

### 2.5. Interlaminar Angle (ILA)

**Figure 3:**
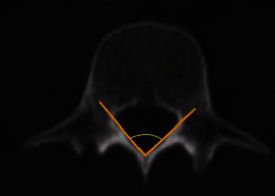
Measurement of interlaminar angle.

Interlaminar angle (ILA) measurment was determined in the axial plane through the upper third of the laminae and defined as the angle between the longitudinal axes of both laminae (Figure 3).

### 2.6. Laminar Inclination (LI)

**Figure 4:**
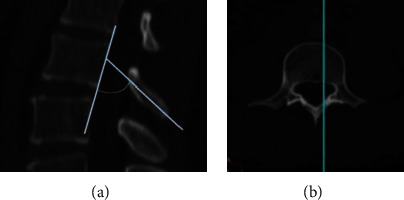
Measurement of laminar inclination (a). (b) demonstrates the location of the sagittal section at the mid lamina.

Measurment of of laminar inclination (LI) was evaluated in the para-sagittal plane and defined as the angle between the longitudinal axis of the mid-lamina and the posterior cortex of the same vertebral body (Figure 4). We have modified the method of Xu et al., as the axis of reference in the current study is the posterior cortex of the vertebra rather than the horizontal plane of the vertebral body [21].

### 2.7. Statistical Analysis

The statistical analyses were calculated via SPSS version 20. The sample size for this study was based on power analysis (*α* = 0.05, *β* = 0.8), and all the metric parameters were checked for normal distribution. Independent *t*-test was used to compare the metric parameters between the study groups for each gender separately. Logistic regression analysis (forward R-L) was also used to determine the variables that associate with DLSS (dependent variable-DLSS, independent variable-vertebral morphometry such as vertebral length and width).

The intraclass correlation (ICC) coefficients were calculated to determine the intratester and intertester reliability of the measurements taken (repeated measurements of 20 individuals). Intratester reliability of the measurements was assessed by one of the authors (JA) who took the measurements twice within intervals of 3-5 days. Intertester reliability involved two testers (JA and KH), who took the measurements within an hour of each other. Both testers were blinded to the results of the measurements. Significant difference was set at *P* < 0.05.

## 3. Results

The intratester and intertester reliability results for all the measurements were high: 0.985 to 0.782 and 0.980 to 0.721, respectively.

### 3.1. Vertebral Body Size in the Study Groups (Control and Stenosis)

The mean values of age and BMI for all the subjects are presented in Table 1.

**Figure 5:**
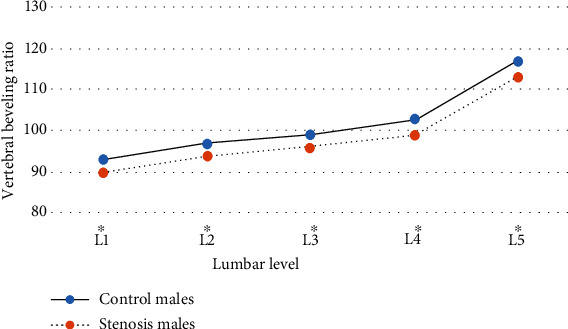
The mean vertebral beveling ratio of the male groups by lumbar level.

**Figure 6:**
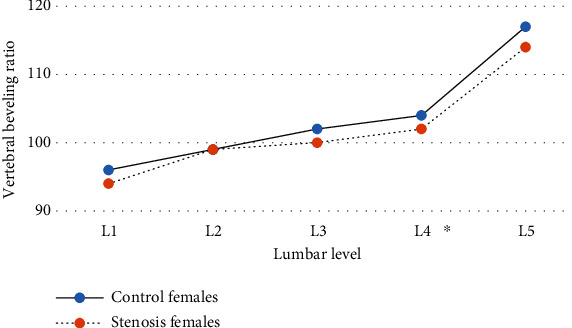
The mean vertebral beveling ratio of the female groups by lumbar level.

The vertebral body length and width values from L1 to L5 levels were significantly greater in both males and females of the stenosis group compared with their counterparts in the control (Table 1). Additionally, vertebral bodies in the stenosis males were more beveled anteriorly at L1 to L3 and less beveled posteriorly at L4 and L5 compared to their counterpart in the control (Figure 5). This implies that the vertebral body in the stenosis group was more kyphotic at L1 to L3 and less lordotic at L4 and L5. Among females, however, significant difference was found only at L4 level (Figure 6).

### 3.2. Bony Canal Diameters in the Study Groups (Control and Stenosis)

The bony AP diameters and CSA values at all lumbar levels were significantly smaller in stenosis males and females compared with their counterparts in the control group (Table 2). However, no significant differences were noted in the ML diameters between the study groups. It is noteworthy that the AP values for almost 50% of the control group were between 17 to 25.4 mm, whereas half of the stenosis group manifested AP values (L2 to L5) between 14.1 and 17 mm (Table 3).

### 3.3. Lamina Measurements in the Study Groups

The mean interlaminar angle values at L4 and L5 levels in the stenosis males were significantly smaller compared to their counterparts in the control. In contrast, these values (L1 to L4) were significantly greater in the stenosis females compared to the control. In addition, the mean lamina inclination values were significantly smaller in the stenosis males (L2-L4) and females (L1-L4) compared to their counterparts in the control group (Table 4).

The logistic regression analysis (adjusted for BMI and age) revealed that VL L1, AP L5, vertebral beveling (L2, L4), and laminar inclination (LI) of L3 were significantly associated with DLSS in males. In females, these are VL of L1, AP (L4, L5), and LI of L1 (Table 5).

## 4. Discussion

The principal results of this study indicate that vertebral morphometry such as vertebral body length (L1), anterior-posterior diameter of bony canal (L5), and laminar inclination increases the likelihood of DLSS development. This result implies that even though the radiological manifestation of DLSS is degenerative process in the three-joint complex, there are certain architectural bony features that could be predisposing factors for the degenerative stenosis.

It is noteworthy that although the AP canal diameters in all lumbar levels were significantly smaller in the DLSS group, the values obtained are still within the normal range of A-P diameters (greater than 12 mm), that was determined previously [8, 22–24]. We also found that almost 50% of the stenosis group manifested AP values (L2 to L5) between 14.1 and 17 mm, whereas half of the control were between 17 and 25.4 mm. This implies that (1) the borderline of the AP value should be modified, and (2) the size of the bony AP diameters has a significant impact on the degenerative spine segment and LSS development.

Verbeist was the first who established the normal and pathologic ranges for vertebral canal dimensions [25, 26]. Later, several studies based on dried skeletal and CT images demonstrated that normal lumbar midsagittal diameter is greater than 12 mm [8, 22–24, 27, 28]. In addition, one study has showed that canal cross-sectional area measurement in CT images was reliable and more sensitive than anterior posterior and interpedicular measurement [29]. Schonstrom el al., however, pointed out via CT study that those individuals who had undergone lumbar decompression manifested normal AP diameter (14.1 mm) [8]. They have also concluded that cross-sectional area of the dural sac is more reliable than bony canal diameters, and the critical size for the dural sac is below 100 mm^2^. Others have reported significant positive correlations between dural sac CSA and walking distance among individuals with spinal stenosis [30, 31]. Zheng and colleagues however have showed that the ratio of CSA of dural sac and spinal canal is a useful method for evaluation of spinal stenosis [9].

The spinal canal size is determined by genetics/or environmental factors that affect the growth of the canal early in life [32]. Pre and perinatal illnesses (e.g., malnutrition and toxins) may significantly stunt the canal growth permanently. Midsagittal diameter is greatly determined before or shortly after birth since by this time, the canal has already exhausted its growing potentials [33]. In contrast, mediolateral (transverse) diameter continues to grow until adulthood [33]. The fact that mediolateral diameters were similar between the study groups (control vs. stenosis) may in some way imply that genetics or early development factors are dominant for determining the spinal canal size in these cases.

We also found that participants in the DLSS group have greater vertebral body dimensions (width and length) compared to control. Furthermore, vertebral body length increases the risk for DLSS development. Indeed, only scant data have been found which indicate a relationship between the vertebral body size with DLSS development. Johns and Thompson (1968) constructed ratios based on spinal canal and vertebral body dimensions in which they identified large and small canal [34]. Zheng and colleagues however revealed that AP diameters of the vertebral body do not provide any evaluation in DLSS [9]. Congenital stenosis was not included in this study, in which the AP canal diameter, pedicle length, and vertebral body width are expected to be smaller in the stenosis group [35]. Abbas et al. (2010) have previously showed that vertebral body dimensions in DLSS were significantly greater from L3 to L5 compared to control [10]. They also argue that one of their elucidation does rely on genetics. More so, our finding is compatible with the recent study that showed greater pedicle width among individuals with DLSS compared to control [11].

Our finding also showed that laminar inclination is significantly associated with DLSS. To the best of our knowledge, very little studies have previously addressed the role of laminar inclination in the development of spinal degenerative changes. The laminar slope angle firstly reported in 1999 and was found to vary across the different vertebral levels [21]. Yu et al. (2021) have recently reported that smaller laminar slope angle is related with LF hypertrophy [36]. The authors also suggest that laminar slope may lead to different changes of the spine unit that ultimately result in DLSS. More so, smaller laminar thoracic slope angle was reported to be associated with greater tension of the ligamentum flavum [37]. Nagaosa and colleagues have also stated that the horizontalization of the lamina and facets is a pathoanatomic risk factor that can predispose the development of degenerative spondylolisthesis [38]. It is noteworthy that the laminar inclination measurement in the current study is unlike the proposed method of Xu et al. (1999) [21] in which they considered the mid horizontal line of the vertebral body as the axis of reference. We opted to use the posterior vertebral border as the axis of reference for measuring the laminar inclination. The posterior cortex of the vertebral body, contrary to the upper endplate, is not prone to any age-related changes such as osteoporotic or degenerative changes of the upper end plate that could influence its orientation relative to the lamina.

Lumbar vertebral size is considered age, gender, race, and level dependent [22, 27, 39, 40]. Contrary to spinal canal diameters, the vertebral body size is associated with degenerative lumbar pathologies [15, 41, 42]. For example, Ruyssen-Witrand et al. (2007) reported in their systematic review study that the smaller vertebral size seems to be at a higher risk of vertebral fracture [42]. In addition, inadequate Ca intake and a high level of physical activities may affect the vertebral body size among women [43, 44]. Oura and colleagues have recently reported that higher BMI is strongly associated with vertebral CSA [45]. Therefore, one could postulate that greater vertebral body size values among the stenosis group relate to anthropometric parameters (e.g., higher BMI) as well as higher impact loading such as heavy manual work [46]. This result supports the notion that DLSS is a multifactorial phenomenon including genetic and environmental factors [47] that may affect both gender in different ways.

We think that the greater vertebral size combined with variation of the laminar inclination and/or AP canal diameters could alter the trajectory of axial loading upon the lumbar spine causing modification in vertebral body beveling. These factors may also harm the spine segment stability leading to different changes of the lumbar spine such as LF thickness, facet joint arthrosis, degenerative listhesis, and osteophyte formation that eventually lead to stenosis.

In summary, although DLSS individuals usually exhibit a degenerative process in the three-joint complex, we assume that they still have unique bony characteristics (e.g., greater vertebral body length and variations of the laminar inclination) that could be a primary and/or a secondary manifestation that could trigger the degenerative changes and the onset of DLSS. We also believe that the AP canal diameter values should be considered for DLSS evaluation.

### 4.1. Limitations of the Study

As this is a retrospective study, no causal relationships are determined between vertebral body size and DLSS. Moreover, large-scale participants in a prospective study are needed to establish whether DLSS individuals have a lesser risk for osteoporotic fractures (due to higher vertebral body size) [42].

## 5. Conclusion

Our study revealed that lumbar bony morphometry such as vertebral body width, AP canal diameter, and laminar inclination had a significant role in the DLSS development.

## Figures and Tables

**Table 1:** Age, BMI and vertebral body length (VL), width (VW), and beveling of the study groups (control vs. stenosis) by lumbar level.

Variables	Males			Females		
	Control (mean ± SD)	Stenosis (mean ± SD)	*P* value	Control (mean ± SD)	Stenosis (mean ± SD)	*P* value
Age (years)	62.8 ± 12	66.2 ± 11	0.066	62 ± 12	62 ± 8	0.800
BMI (kg/m^2^)	27.3 ± 4	28.9 ± 4	0.021	27.6 ± 5	31.4 ± 5	<0.001
VL L1 (mm)	29.9 ± 2.7	31.3 ± 2.9	0.003	26.4 ± 2.1	27.8 ± 2.1	<0.001
VL L2 (mm)	31.1 ± 2.2	32.6 ± 2.8	<0.001	24.5 ± 2.2	28.7 ± 1.9	<0.001
VL L3(mm)	32.6 ± 2.3	34 ± 2.5	<0.001	29.3 ± 2.3	30.9 ± 2.5	<0.001
VL L4 (mm)	32.8 ± 2.3	34.1 ± 2.6	0.001	30 ± 2.4	31.7 ± 2.2	<0.001
VL L5 (mm)	32.9 ± 2.3	34.5 ± 2.7	<0.001	30.5 ± 2	32.1 ± 2.4	<0.001
VW L1 (mm)	39.7 ± 2.9	40.9 ± 3.1	0.009	35 ± 2.4	36.1 ± 2.4	0.006
VW L2 (mm)	40.9 ± 2.8	42.8 ± 3.5	<0.001	36.8 ± 2.6	37.9 ± 2.3	0.003
VW L3 (mm)	42.8 ± 2.9	44.8 ± 3.1	<0.001	38.8 ± 2.8	40.1 ± 2.6	0.002
VW L4 (mm)	44.7 ± 2.9	46.6 ± 3.2	<0.001	40.8 ± 2.8	42.2 ± 8	0.001
VW L5 (mm)	49.1 ± 3.8	51.3 ± 5	0.002	45 ± 3.4	47.1 ± 3.3	<0.001

BMI: body mass index; SD: standard deviation.

**Table 2:** Vertebral bony canal diameters (AP, ML, and CSA) of the study groups (control vs. stenosis) by lumbar level.

Variables	Males			Females		
	Control (mean ± SD)	Stenosis (mean ± SD)	*P* value	Control (mean ± SD)	Stenosis (mean ± SD)	*P* value
AP L1	18.7 ± 1.4	17.8 ± 1.4	<0.001	18.7 ± 1.4	17.6 ± 1.4	<0.001
AP L2	17.8 ± 1.6	16.6 ± 1.5	<0.001	18 ± 1.6	16.6 ± 1.3	<0.001
AP L3	16.9 ± 1.7	15.4 ± 1.5	<0.001	17.3 ± 1.5	15.5 ± 1.5	<0.001
AP L4	17.6 ± 1.9	15.6 ± 1.6	< 0.00	18 ± 1.6	15.5 ± 1.6	<0.001
AP L5	18.8 ± 2.2	16.1 ± 1.9	<0.001	18.8 ± 2.2	15.9 ± 1.8	<0.001
ML L1	25.5 ± 1.8	25.6 ± 1.8	0.725	24.5 + 2.4	24.2 ± 2.2	0.479
ML L2	26 ± 2.1	26.2 ± 2.1	0.478	25.4 ± 2.5	24.7 ± 2.1	0.086
ML L3	27 + 2.2	27.3 ± 2.6	0.526	26.5 ± 2.4	25.9 + 2.2	0.064
ML L4	28.6 ± 3	29.4 ± 4.1	0.141	28.7 ± 2.7	28.6 ± 2.9	0.663
ML L5	34.9 ± 3.9	35.3 ± 4.9	0.566	34.8 ± 4.1	34.3 ± 3.9	0.483
CSA L1	296 ± 41	274 ± 36	<0.001	285 ± 42	256 ± 36	<0.001
CSA L2	283 ± 40	256 ± 39	<0.001	283 ± 45	243 ± 34	<0.001
CSA L3	279 ± 45	252 ± 41	<0.001	282 ± 74	236 ± 38	<0.001
CSA L4	305 ± 52	268 ± 53	<0.001	308 ± 51	253 ± 39	<0.001
CSA L5	370 ± 66	310 ± 59	<0.001	374 ± 70	297 ± 57	<0.001

AP: anterior-posterior; ML: mediolateral; CSA: cross-section area; SD: standard deviation.

**Table 3:** Percentage of subjects with AP diameters between 12.1-14 mm, 14.1-17 mm, and > 17 mm, in the studied groups by levels.

Levels	Control group (*n* = 180)			Stenosis group (*n* = 165)		
	12.1-14 mm	14.1-17 mm	> 17 mm	12-14 mm	14.1-17 mm	> 17 mm
L1	0	13	87	0	32.1	67.1
L2	0	31.7	68.3	4.8	58	36.6
L3	1.7	50	48.3	17	67	15.8
L4	1.1	32.8	66.1	17.6	63	18.8
L5	0.6	22.2	80	13.9	59.8	22.8

**Table 4:** Interlaminar angle (ILA) and laminar inclination (LI) of the study groups (control vs. stenosis) by lumbar level.

Variables	Males			Females		
	Control (mean ± SD)	Stenosis (mean ± SD)	*P* value	Control (mean ± SD)	Stenosis (mean ± SD)	*P* value
ILA L1	116 ± 10	116 ± 9	0.889	114 ± 10	117 ± 11	0.111
ILA L2	116 ± 9	114 ± 10	0.265	112 ± 9	117 ± 9	0.002
ILA L3	113 ± 11	111 + 11	0.291	108 ± 10	116 ± 12	<0.001
ILA L4	102 ± 11	98 ± 12	0.025	98 ± 11	104 ± 11	0.001
ILA L5	98 ± 11	90 ± 13	<0.001	94 ± 10	92 ± 11	0.225
LI L1	22.6 ± 5	21.4 ± 5	0.120	26.5 ± 4	23.7 ± 4	<0.001
LI L2	26.4 ± 6	23.7 ± 5	0.003	29.5 ± 5	26.5 ± 4	<0.001
LI L3	31.4 ± 6	27.5 ± 4	<0.001	34 ± 5	29 ± 5	<0.001
LI L4	39.5 ± 6	36.7 ± 7	0.001	42.3 ± 6	37.8 ± 6	<0.001
LI L5	46.7 ± 8	47.4 ± 7	0.599	49.8 ± 6	50.4 ± 6	0.518

SD: standard deviation.

**Table 5:** Variables that significantly associate with degenerative lumbar spinal stenosis (males and females listed separately).

	OR	(CI) 95%	*P* value
Males			
BMI	1.135	1.027-1.256	0.013
Age	1.066	1.022-1.629	0.002
VL L1	1.273	1.094-1.481	0.002
AP L5	0.474	0.364-0.618	<0.001
Beveling L2	0.919	0.852–0.991	0.028
Beveling L4	0.930	0.873-0.990	0.023
LI L3	0.901	0.822-0.987	0.025
Females			
BMI	1.106	1.018-1.201	0.017
Age	1.056	1.010–1.105	0.016
VL L1	1.473	1.164-1.864	0.001
AP L4	0.490	0.327-0.734	0.001
AP L5	0.664	0.493-0.895	0.007
LI L1	0.856	0.761-0.963	0.010

VW: vertebral width; AP: anterior-posterior; LI: laminar inclination; VL: vertebral length.

## Data Availability

The data used to support the findings of this study are available from the corresponding author upon request.
